# Utility of Magnetic Bead-Based Automated DNA Extraction to Improve Chagas Disease Molecular Diagnosis

**DOI:** 10.3390/ijms26030937

**Published:** 2025-01-23

**Authors:** Priscila S. G. Farani, Jacqueline Lopez, Amanda Faier-Pereira, Alejandro Marcel Hasslocher-Moreno, Igor C. Almeida, Otacilio C. Moreira

**Affiliations:** 1 Department of Biological Sciences, The University of Texas at El Paso, El Paso, TX 79902, USA; 2 Platform of Molecular Analysis, Laboratory of Molecular Virology and Parasitology, Oswaldo Cruz Institute, Oswaldo Cruz Foundation, Rio de Janeiro 21040-360, RJ, Brazil; 3 Evandro Chagas National Institute of Infectious Diseases, Oswaldo Cruz Foundation, Rio de Janeiro 21040-360, RJ, Brazil

**Keywords:** *Trypanosoma cruzi*, qPCR, chagas disease, DNA extraction

## Abstract

Chagas disease, caused by *Trypanosoma cruzi*, remains a significant global health challenge, particularly in the molecular diagnostics of low parasitemia during the chronic phase. This highlights the critical need for enhanced diagnostic methodologies. In response, this study evaluates the effectiveness of an automated magnetic beads-based DNA extraction method in improving the molecular diagnosis of Chagas disease compared to the traditional silica column-based extraction. Accordingly, this research seeks to enhance the DNA yield, purity, and sensitivity of real-time PCR (qPCR) assays for detecting *T. cruzi* satDNA. Blood samples spiked with guanidine–EDTA solution and varying concentrations of *T. cruzi* were used to compare the two extraction methods. The results indicated that the magnetic bead-based method outperformed the silica column in terms of DNA concentration, purity, and earlier detection of *T. cruzi* satDNA. Although both methods had similar limits of detection at a 95% confidence interval, the magnetic bead-based approach demonstrated higher sensitivity and reproducibility, particularly in low-parasitemia samples. The findings suggest that the magnetic beads-based DNA extraction method offers a more reliable, faster, and more sensitive alternative for diagnosing chronic Chagas disease, potentially improving clinical outcomes by enabling more accurate and earlier parasite detection.

## 1. Introduction

Chagas disease (CD), also known as American trypanosomiasis, is caused by the protozoan parasite *Trypanosoma cruzi* [1] and is primarily transmitted through contact with the feces or urine of the triatomine bug. Approximately 6 million people worldwide are infected with this debilitating disease. While vector-borne transmission remains the most common route, particularly prevalent in Mexico, Central, and South America, CD can also be transmitted through various other means, including blood transfusion, congenital transmission, solid organ or bone marrow transplantation, and oral ingestion of uncooked food and water and juices contaminated by infected triatomine vectors [2]. Traditionally, CD was confined to rural areas of Latin America, where vectorial transmission was the predominant route of infection. However, the shifting demographics of rural-to-urban migration have facilitated the spread of CD to non-endemic regions through congenital transmission and blood donations [3]. Travelers and immigrants have emerged as parasite carriers, amplifying its significance as a global public health and medical concern [4].

Chagas disease typically follows a biphasic clinical course, consisting of an acute phase followed by a chronic phase. The acute phase, which can manifest at any age, is typically asymptomatic and persists for 4–8 weeks, with parasitemia gradually waning over approximately 90 days. Conversely, 20–30% of patients in the chronic phase may experience organ involvement, often appearing a decade or more following the initial acute infection [3]. Diagnosing CD during the acute phase primarily relies on detecting the presence of the parasite in the blood. As time progresses, parasitic levels in the bloodstream diminish significantly, rendering them mostly undetectable or transient within a few months [5]. During the chronic phase, due to low parasitemia, diagnosis predominantly relies on serological methods [6]. However, serological diagnosis can be problematic, as it often yields inconsistent results across different tests and does not exhibit negative seroconversion following antiparasitic treatment. This makes evaluating treatment efficacy particularly challenging. Thus, molecular techniques, such as quantitative real-time PCR (qPCR), have become invaluable tools in complementing CD diagnosis. These techniques enable the quantification of *T. cruzi* parasite load throughout the clinical course of the disease and primarily serve as markers of treatment failure [7,8,9]. Moreover, recent advances in molecular diagnostics have facilitated the development of highly sensitive methods for detecting and quantifying *T. cruzi* satellite DNA (satDNA) via qPCR. Implementing qPCR assays for parasite load determination has yielded promising results when applied to blood samples from infected patients [10,11]. Nevertheless, quality control in molecular techniques for diagnosing CD is essential for ensuring a reliable diagnosis [8]. To develop an accurate laboratory technique for diagnosing CD, addressing the low and intermittent number of parasites circulating in the blood during the chronic phase is crucial [12]. Thus, while qPCR has been utilized to detect and quantify parasites in clinical samples, its varied analytical accuracy, specificity, and sensitivity pose challenges in standardizing the technique for routine clinical use [13].

Furthermore, due to the extensive genomic and proteomic diversity among and within different parasite genotypes and the variability in human antibody responses across diverse endemic and non-endemic regions, diagnosing CD remains a significant and pressing challenge, particularly during its chronic stage [14,15,16]. Recent advancements in molecular diagnostics have greatly improved the detection and management of CD. qPCR has gained international consensus as a valuable tool for the molecular diagnosis of CD, with its effectiveness validated through multicenter studies [10,12]. These advancements have improved diagnostic accuracy and facilitated the monitoring of patients undergoing antiparasitic treatment. Clinical studies such as TESEO [17], MULTIBENZ [18,19], and BENEFIT [15] have demonstrated the utility of qPCR and PCR in identifying therapeutic failures. However, neither method can be reliably used to assess cures due to their relatively low–medium sensitivity in detecting residual parasitemia. Moreover, developing and validating in vitro diagnostic (IVD) kits [20,21] has further propelled the field. These kits have been rigorously tested and validated for human diagnosis, offering high sensitivity and specificity. However, despite these advances, limited progress has been made in pre-PCR methodologies, including all steps from sample stabilization to the extraction and acquisition of high-quality DNA. Enhancements in these areas could improve the sensitivity and reproducibility of CD molecular diagnosis.

Continued advancements in diagnostic tools are essential to overcoming the existing limitations, ensuring more accurate disease detection and effective treatment monitoring. This progress will ultimately lead to improved patient outcomes and more effective public health interventions [22]. Thus, in the present study, we validate using an automated magnetic bead-based DNA extraction method compared to the traditional silica-based column method. We aim to improve the reproducibility, yield, and quality of DNA extraction from blood samples and enhance the sensitivity of parasite DNA detection by qPCR. This approach offers a hands-free process that saves time and reduces the risk of cross-contamination.

## 2. Results

### 2.1. DNA Quality and Sensitivity of T. cruzi satDNA Amplification from DNA Extracted with Silica Column and Magnetic Beads Purification Methods

**Figure 1:**
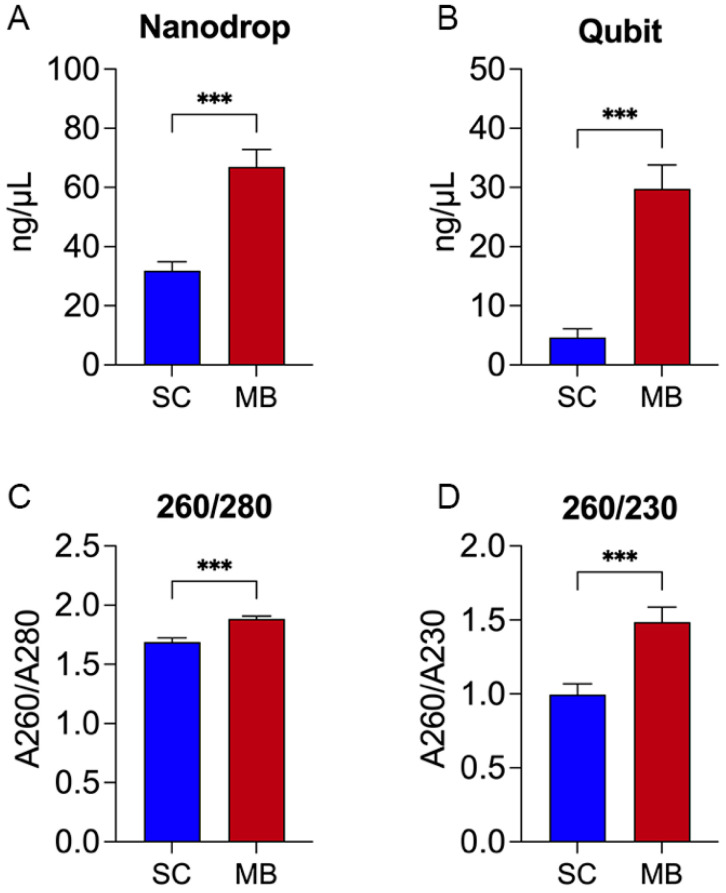
DNA extraction yields and purity ratios for silica column and automated magnetic bead purification systems. DNA was extracted from GEB samples using either the silica column-based (SC) method (blue) or the automated magnetic bead-based (MB) method (red) and quantified using (**A**) NanoDrop or (**B**) Qubit. Purity was assessed through (**C**) 260/280 and (**D**) 260/230 ratios using NanoDrop. DNA concentration (ng/μL) is presented as the mean ± SD for each group. Statistical significance was determined using one-way ANOVA with pairwise multiple comparisons (***, *p* < 0.001).

First, aliquots of GEB were spiked with different concentrations of *T. cruzi*, ranging from 10^4^ to 10^−5^ Par. Eq./mL, and ExoIPC. Subsequently, DNA was isolated by a silica column (SC) or magnetic beads (MBs) purification system (Supplementary Figure S1). To assess the yield of each purification method, DNA was quantified by a NanoDrop spectrophotometer (Figure 1A) and Qubit fluorometer (Figure 1B), showing statistically higher concentrations for the MBs nucleotide extraction (NanoDrop: 66.92 ± 5.98 ng/μL; Qubit: 29.75 ± 4.07 ng/μL) methods when compared to SC (NanoDrop: 31.88 ± 2.98 ng/μL; Qubit: 4.65 ± 1.48 ng/μL). Similarly, the nucleic acid purity ratios determined by the absorbance ratio at the wavelengths 260/280 and 260/230 nm showed significantly higher ratios for the MBs extraction method for both 260/280 nm (MB: 1.88 ± 0.02; SC: 1.69 ± 0.03) (Figure 1C) and 260/230 (MB: 1.48 ± 0.10; SC: 0.99 ± 0.07) than the SC extraction method (Figure 1D). These results indicate that the MBs method yields a higher concentration of DNA and significantly improved purity compared to the SC method.

Additionally, the isolated DNA was subjected to qPCR assessment for RNAseP (Supplementary Figure S2), which further confirmed the superior recovery of human DNA in the samples processed using the MBs method. This is reflected by lower Ct mean values (RNAse P Ct mean: 19.54 ± 0.086, with a threshold of 0.01; Ct mean: 20.41 ± 0.11, with a threshold of 0.02) compared to the SC method (RNAse P Ct mean: 22.17 ± 0.19, with a threshold at 0.01; Ct mean: 23.05 ± 0.20, with a threshold of 0.02). Furthermore, a comparison between the thresholds of 0.01 (Supplementary Figure S2A and Table S1) and 0.02 (Supplementary Figure S2B and Table S2) revealed no statistically significant difference (Supplementary Figure S2C). Accordingly, subsequent quantification analysis was conducted using the threshold of 0.01 for consistency in further assessments.

**Figure 2:**
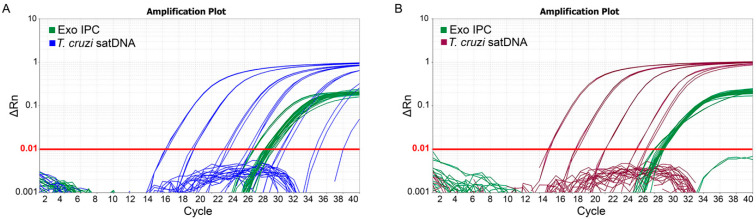
Quantitative real-time PCR for Exogenous Internal Positive Control (ExoIPC). (**A**) and *T. cruzi* satDNA (**B**). The amplification plot for ExoIPC (green curves) and *T. cruzi* satDNA (blue or purple curves) for (**A**) SC method and (**B**) automated MB purification system.

Purified DNA was then subjected to qPCR for *T. cruzi* satDNA and ExoIPC assessment, comparing the SC and MBs extraction methods (Figure 2). Table 1 presents the Ct mean values for each target and method. ExoIPC exhibited consistent Ct values across all curve points for the SC (29.49 ± 1.46) and MBs (29.92 ± 2.79) methods, indicating accuracy in the isolation process for both approaches. *T. cruzi* satDNA was also consistent for both methods, though the MBs method demonstrated lower Ct values (15.17 ± 0.12 to 34.51 ± 0.00) compared to the SC (16.48 ± 0.14 to 32.78 ± 3.30) method, indicating more efficient detection with the magnetic beads. It is important to note that both methods failed to amplify the *T. cruzi* satDNA below 10^−2^ Par. Eq./mL, a known limitation of this qPCR method [23]. However, at 10^0^ Par. Eq./mL, the ΔCt (Ct_SC_ − Ct_MB_) was 4.87, indicating that at this concentration, the automated MBs purification method detected approximately 29-fold more satDNA than the SC method for the same sample concentration. It is important to note that at 10^4^ Par. Eq./mL, a significant increase in Ct values for ExoIPC was observed (Ct 33.57 ± 2.76 for the SC method, and 38.08 ± 0.41 for the MBs method), likely due to the robust amplification of the *T. cruzi* satDNA in multiplex. A moderate increase in Ct values was also observed at 10^3^ Par. Eq./mL.

### 2.2. T. cruzi satDNA Standard Curve Assessment in Silica Column and Magnetic Beads Purification Methods for Determination of Linearity

**Figure 3:**
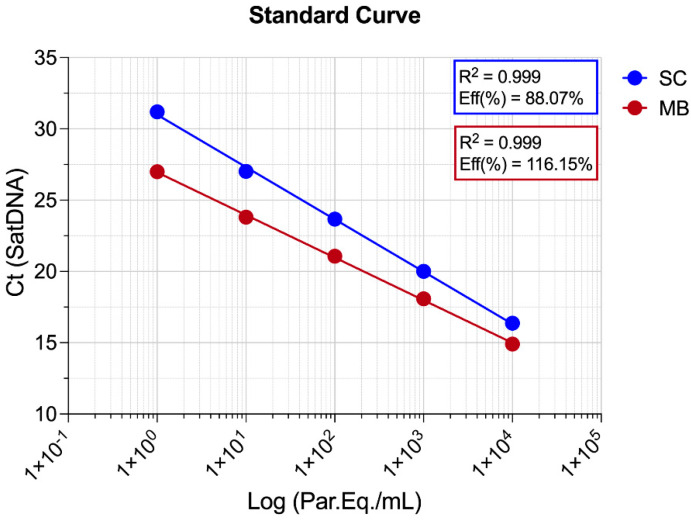
Standard curves for *T. cruzi* satDNA. Standard curves for silica column-based (SC) and magnetic beads-based (MBs) method. Samples were analyzed in triplicate, and the results are shown as mean ± SD for each group.

For this assay, GEB was spiked with *T. cruzi* to reach 10^4^ par/mL, followed by DNA purification using both methods tested in this study. After purification, the DNA from *T. cruzi*-spiked GEB was subjected to 1:10 serial dilution in negative DNA (from non-spiked GEB), ranging from 10^4^ to 10^−5^ Par. Eq./mL (Supplementary Figure S3). Quantification of DNA yield from both methods was performed by the NanoDrop One C spectrophotometer (Thermo Fisher Scientific, Waltham, MA, USA) (Supplementary Figure S4A) and Qubit fluorometer (Supplementary Figure S4B), showing statistically higher concentrations for the magnetic beads’ nucleotide extraction (NanoDrop: 149.70 ± 1.62 ng/μL; Qubit: 83.60 ± 12.69 ng/μL) methods when compared to silica column (NanoDrop: 15.55 ± 1.61 ng/μL; Qubit: N/A). Similarly, the nucleic acid purity ratios determined by the absorbance ratio at wavelengths of 260/280 and 260/230 nm showed higher ratios for the magnetic beads’ extraction method for both 260/280 nm (MB: 1.90 ± 0.00; SC: 1.69 ± 0.03) (Supplementary Figure S4C) and 260/230 nm (MB: 2.01 ± 0.03; SC: 0.92 ± 0.06) (Supplementary Figure S4D). Following the qPCR assay, standard curves for the detection of satDNA demonstrated a dynamic range from 10^4^ to 1 Par. Eq./mL, with a coefficient of determination (r^2^) of 0.999 for both methods. However, the SC isolation method exhibited an efficiency of 88.07%, while the MBs purification system achieved 116.15% (Figure 3). Additionally, across all standard curve points, higher Ct values were consistently observed for the SC isolation method compared to the MBs method.

### 2.3. Determination of Limit of Detection (LOD_95_) for DNA Samples Purified Using Silica Column or Magnetic Beads

To evaluate the limit of detection (LOD) for both DNA extraction methods, guanidine EDTA blood (GEB) was spiked with known concentrations of *T. cruzi* trypomastigotes. The spiked GEB was then subjected to a two-fold serial dilution in non-spiked GEB to produce a range of concentrations, starting from 20 parasite equivalents (Par. Eq.)/mL down to 0.020 Par. Eq./mL. For each dilution, 2 mL of sample was used. Each sample underwent three independent extractions with both methods to account for variability and ensure robustness (Supplementary Figure S5). Quantitative PCR analysis was performed on all extracted samples, with 12 replicates for each dilution point to achieve statistical significance. Fluorescence data from qPCR were analyzed to determine the cycle threshold (Ct) values, which were used to identify the lowest concentration at which 95% of replicates tested positive (LOD95). The experimental design was guided by the principles outlined in Burd et al. 2010 [24], which recommends a systematic approach for determining the LOD in molecular assays. This reference provided a framework for ensuring rigor and reproducibility in our LOD assay, particularly regarding the use of multiple replicates, serial dilutions, and independent extractions to accurately establish assay sensitivity.

**Figure 4:**
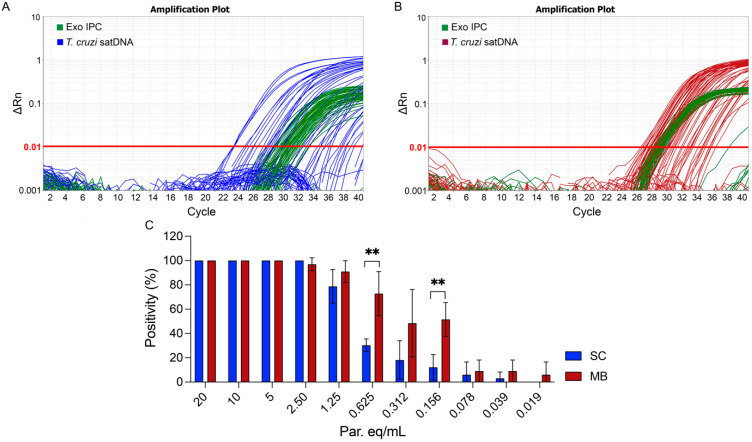
Quantitative real-time PCR amplification plots for ExoIPC and *T. cruzi* satDNA in the limit of detection (LOD) assay. The amplification plots display ExoIPC (green curves) and *T. cruzi* satDNA (blue and purple curves) for both (**A**) the SC-based method and (**B**) the automated MBs purification system. (**C**) Comparison of PCR positivity between SC and MBs extraction methods. Statistical significance was determined using one-way ANOVA with pairwise multiple comparisons (**, *p* < 0.01).

**Figure 5:**
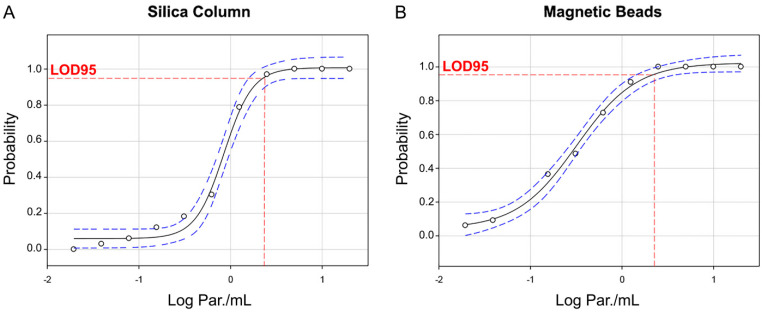
Probit regression (dose–response analysis) for the 95% limit of detection (LOD95) comparison between DNA extracted by silica column or magnetic beads automated method. LOD95 assay for *T. cruzi* satDNA detection using DNA extracted by (**A**) the SC method and (**B**) the MBs method. DNA extracted from each GEB sample with different parasite loads was evaluated in 33 technical replicates. The horizontal red dotted line corresponds to the estimate of LOD parameters with a confidence interval (CI) of 95%. The black curves represent the probit sigmoid dose–response curves, and the blue dashed lines correspond to the 95% CI. LOD_95_ SC = 2.29 Par. Eq./mL and LOD_95_ NB = 2.09 Par. Eq./mL.

Amplification plots for the SC (Figure 4A) and MBs (Figure 4B) purification methods showed notable differences, particularly in the standard deviation values for ExoIPC. The MBs method (28.93 ± 0.67) demonstrated greater consistency and reproducibility than the SC method (29.60 ± 1.00). Furthermore, the qPCR targeting satDNA exhibited higher positivity to low parasitic loads (0.625, 0.312, and 0.156 Par. Eq./mL) when using DNA extracted with the MBs method compared to the SC method (Figure 4C). In 33 technical replicates across a range of 20 to 0.019 Par. Eq./mL, the MBs method consistently showed higher PCR positivity at every concentration tested, detecting at least two replicates (2/33) at the lowest concentration of 0.019 Par. Eq./mL, whereas the SC method detected none (0/33). This suggests the superior sensitivity of the automated MB purification method in detecting *T. cruzi* satDNA (Supplementary Table S3). Probit regression analysis indicated a 95% LOD (LOD_95_) of 2.29 Par. Eq./mL for the SC method (Figure 5A) and 2.09 Par. Eq./mL for the MBs method (Figure 5B). Although the LOD_95_ values for both methods were similar, the MBs method was able to detect lower concentrations of *T. cruzi* satDNA, further suggesting a higher sensitivity with this method.

### 2.4. Reproducibility of T. cruzi satDNA Detection in DNA Samples Purified Using Silica Column or Magnetic Beads

**Figure 6:**
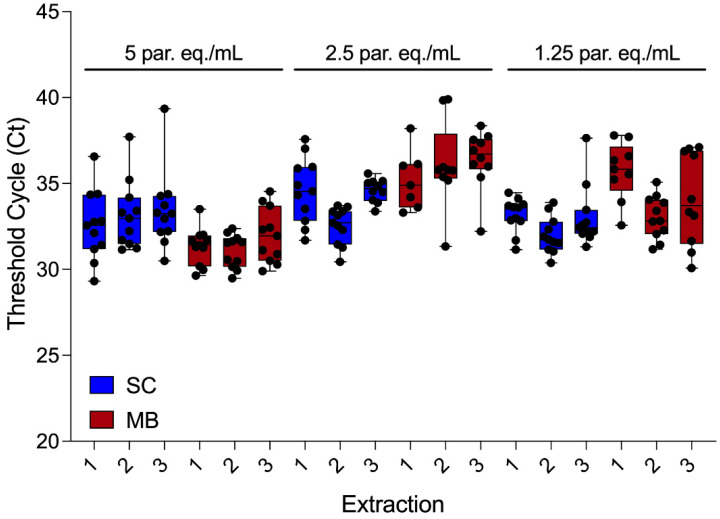
Reproducibility of qPCR assays targeting *T. cruzi* satDNA. GEB aliquots spiked with varying concentrations of *T. cruzi* (5.0, 2.5, and 1.25 Par. Eq./mL) were tested in 33 technical replicates to assess reproducibility at each concentration.

To compare the reproducibility of the *T. cruzi* satDNA detection in DNA samples purified by both methodologies, the Ct values were analyzed for each assay at concentrations of 5, 2.5, and 1.25 Par. Eq./mL, representing parasitic loads around the LOD_95_ (Figure 6). Table 2 summarizes the number of positive samples, mean Ct values, and coefficient of variation for each concentration, comparing both purification methods. The MBs method yielded a higher number of positive samples across all concentrations compared to the SC method. Additionally, Ct values were consistently lower with the MBs method, indicating that *T. cruzi* satDNA was detected earlier in the amplification plot. Despite these differences, the coefficient of variation remained similar between the two methods. Notably, for the MBs method, the coefficients of variation were below 5.0% for two of the three concentrations tested (5.0 and 2.5 Par. Eq./mL), demonstrating a high reproducibility, especially at or near the LOD_95_.

### 2.5. Comparison of DNA Extraction Methods in GEB Samples from Patients with Chronic Chagas Disease

**Figure 7:**
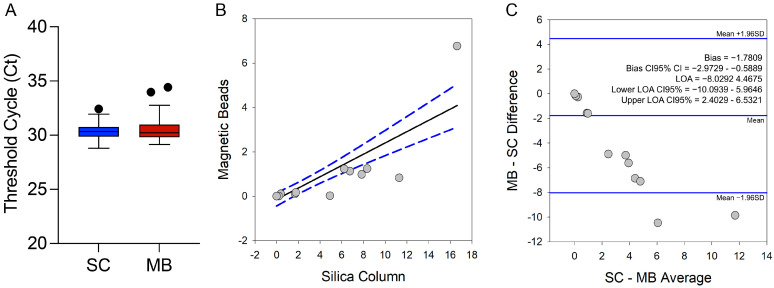
Agreement of parasite load quantification in DNA extracted using silica column or magnetic beads method. (**A**) Box plot showing the Ct values for the ExoIPC in 40 GEB samples from chronic CD patients. Dots outside the box indicate outlier values. (**B**) Scatter plot comparing parasite load between the SC and MBs methods. The black line represents the linear regression, while the blue dashed lines represent the 95% confidence interval (CI). (**C**) Bland–Altman bias (difference) plot analyzing the agreement between parasite load quantification in DNA extracted using the silica column and magnetic beads methods. Bias, standard deviation, and limits of agreement are shown on the right. The central horizontal blue line represents the mean difference, and the outer horizontal blue lines represent the limits of agreement. In both (**B**,**C**), gray dots represent the parasite load of each patient. LOA, limit of agreement; CI_95%_, confidence interval at 95%.

The performance of the DNA extraction using the MBs method was evaluated against the SC extraction method to assess its effectiveness in diagnosing CD in a cohort of chronic CD patients from Brazil. The amplification of ExoIPC in GEB samples from patients was compared between the two DNA extraction methods. The MBs method produced Ct values similar to the SC method, with no statistically significant difference (Figure 7A).

Table 3 summarizes the sensitivity and specificity of the qPCR assay using DNA extracted by both methods. In CD patients (positive serology, *n* = 30), *T. cruzi* DNA was detected in 11 out of 30 samples, yielding a sensitivity of 36.7%. This consistency between the two methods indicates that both are equally effective in detecting the parasite in serologically positive samples. Among serologically CD-negative individuals (*n* = 10), both methods showed perfect agreement, with no false positives, resulting in 100% specificity for both extraction techniques. The total number of qPCR-positive samples was 11 for both methods. In contrast, 29 samples were qPCR-negative, further highlighting the comparable sensitivity and specificity of the SC and MBs extraction methods. These findings confirm that both methods are reliable for the molecular diagnosis of CD, providing consistent performance in detecting *T. cruzi* satDNA in clinical samples.

Next, we illustrate the agreement between parasite loads quantified using the two DNA extraction methods (Figure 7A), where the close alignment of the data points along the regression line indicates a strong correlation between the results of both methods (Figure 7B). Additionally, the Bland–Altman analysis assessed the agreement between parasite loads in DNA from the two extraction methods (Figure 7C), revealing high concordance, with a narrow range between the limits of agreement (from −8.03 to 4.47) and a small bias (−1.78). These results further validate the MBs method as reliable, offering similar sensitivity and accuracy for *T. cruzi* satDNA detection compared to the traditional SC method.

## 3. Discussion

Over the last decade, qPCR has become increasingly important in diagnosing Chagas disease due to its enhanced sensitivity and specificity in detecting the presence of *T. cruzi* DNA in blood samples from acute and chronic stages. It enables the quantification of parasite DNA, offering a valuable tool for monitoring treatment efficacy [25]. However, few advances have been made in the pre-PCR stage, which includes sample stabilization and DNA extraction. In this study, we compared two DNA purification methods: one using a widely utilized silica column DNA isolation kit and a newer method based on magnetic beads with an automated system. The latter is claimed to provide higher-quality DNA, reduce contamination, and enhance the reproducibility of molecular assays. Using guanidine–EDTA (GEB) to stabilize blood samples is critical for the molecular diagnosis of Chagas disease, particularly when transporting samples from the field to the laboratory at room temperature. GEB preserves DNA integrity by preventing degradation, ensuring accurate diagnostic results [26]. However, the compatibility of GEB with different DNA extraction methods is a potential concern. While GEB effectively stabilizes samples, it can cause PCR inhibition when used with certain magnetic bead-based extraction kits. This incompatibility arises from guanidine hydrochloride in GEB, a potent PCR inhibitor affecting DNA extraction efficiency and subsequent amplification. Therefore, it is crucial to consider the compatibility of GEB with the selected DNA extraction method to prevent compromised diagnostic outcomes.

In our initial assessment, we observed significant differences in the extraction yields between the two methods, with the magnetic beads method demonstrating superior performance in both DNA quantity and purity. This was further confirmed by the detection of human DNA (RNAse P gene) in the blood samples, where the average Ct values were consistently 2–3 cycles lower with the magnetic beads method. This suggests the earlier detection of *T. cruzi* DNA, likely due to the higher total DNA concentration in the samples extracted using the magnetic beads approach. Utilizing a DNA purification method that yields a higher quantity of DNA offers significant potential for improving the assessment of samples from chronic Chagas disease patients, where parasite DNA levels are often intermittent and difficult to detect [12,27]. This increased DNA yield was further validated by the detection of *T. cruzi* satDNA, where the magnetic beads method demonstrated nearly 32 times higher detection, based on Ct values, at a concentration of 100 Par. Eq./mL compared to the silica column method. Furthermore, standard curves for satDNA were generated for both methods, yielding an R^2^ of 0.999. These results are consistent with previous studies that demonstrated linearity greater than 0.98 for the same qPCR assay used in this study [23]. Additionally, earlier research reported an efficiency of approximately 77.5% for this TaqMan reaction when using silica column purification [10], which is lower than the efficiencies observed in this study for the silica column (88.1%) and magnetic beads (116.2%) purification methods.

In our exploration to determine the limit of detection of our assays, although the LOD_95_ of both methods showed minimal variation, we observed a higher number of positive samples when the parasite load was below 0.625 Par. Eq./mL using the magnetic beads purification method. This result enables the improved detection of *T. cruzi* DNA, particularly in cases of exceptionally low parasitemia, which is common in chronic Chagas disease patients. A recent study advocating for best practices in the molecular diagnostics of Chagas disease recommended a minimum of three serial blood samples per patient, with three DNA extractions. This approach is designed to enhance qPCR sensitivity and positivity rates, particularly during the recruitment process for clinical trials involving chronic patients, where it is critical to assess treatment failure effectively [27].

One of our objectives in comparing the nucleotide purification methods was to reassess the necessity of performing three independent DNA extractions and qPCR assessments for each patient. While we performed three DNA extractions per method for comparative purposes, the aim was to evaluate the performance of the automated extraction method relative to the column-based extraction method, regardless of the number of extractions. For patient samples, we conducted a single DNA extraction to maintain consistency with the standard diagnostic workflow. Studies suggest that qPCR sensitivity can increase when multiple DNA extractions are performed from the same guanidine–EDTA blood sample, particularly in chronic patients [27]. However, for acute patients, due to the limited availability of samples and the already high sensitivity of parasitological and qPCR methods, there is no evidence indicating a relationship between the number of extractions and improved qPCR sensitivity, as this approach is generally unnecessary.

Moreover, the genetic variability of *T. cruzi* poses another critical challenge to molecular diagnostics, as the parasite exhibits extensive genetic diversity, categorized into seven discrete typing units (DTUs, TcI-TcVII). This diversity could influence the sensitivity and specificity of molecular assays, as differences in target sequence conservation across DTUs may lead to variable detection efficiencies, as demonstrated by Ramirez et al. (2015) when designing qPCR assays for multiple *T. cruzi* DTUs, highlighting the importance of robust diagnostic tools that can detect diverse strains effectively [12]. Similarly, Burgos et al. (2017) emphasized the need for assays adaptable to genetic variations, particularly in regions with high DTU heterogeneity [28]. This underscores the importance of validating molecular diagnostic methods across all major DTUs to ensure the reliable detection and accurate quantification of *T. cruzi* DNA, regardless of strain variability. Our study further supports this rationale, as the superior sensitivity observed with the magnetic beads method demonstrates its potential to enhance the detection of *T. cruzi* across genetically diverse populations represented by the clinical samples validated in this study.

Implementing the magnetic beads purification method could offer significant advantages for molecular laboratories by reducing time and reagent consumption. Our results demonstrated that the magnetic beads method not only increased positivity rates but also enhanced reproducibility. The qPCR positivity rate using the magnetic beads method was significantly higher than the silica column method, at parasitemia levels of 0.625 and 0.156 Par. Eq./mL. This improvement in positivity is critical for the accurate diagnosis and monitoring of treatment efficacy in chronic CD patients, where low parasitemia often leads to false negatives. Furthermore, we highlighted the superior reproducibility of the magnetic beads method, with consistent results across multiple tests, which is essential for reliable longitudinal studies and clinical trials. This consistency minimizes variability in the test results, ensuring that *T. cruzi* DNA detection remains both reliable and reproducible across different sample batches and testing conditions.

However, the anticipated increase in qPCR sensitivity using DNA extracted by magnetic beads was not observed for chronic patients with GEB samples. There was 100% agreement between the positive and negative results for DNA extracted by both methods, along with strong agreement in parasite load quantification, as demonstrated by the Bland–Altman analysis. Despite this, the magnetic beads extraction method offers notable advantages, including faster processing, greater automation, and reduced risk of cross-contamination and human error. Additionally, while the cost difference between silica column and magnetic bead kits is minimal, the magnetic beads method requires an upfront investment in an extraction robot. However, more economical options for automated DNA extraction, such as modified 3D printers adapted for nucleic acid extraction, are available [29]. In this study, only one DNA extraction was performed per GEB sample; performing multiple extractions may better demonstrate the increased sensitivity of molecular diagnosis for Chagas disease achieved through magnetic beads extraction. We plan to investigate this further in a future study.

## 4. Materials and Methods

### 4.1. Cell Culture

LLC-MK2 cells (ATCC Cat n. CCL-7) were infected with *T. cruzi* trypomastigotes of the CL Brener clone (discrete typing unit (DTU) VI) [14] at a multiplicity of infection (MOI) of 10:1, in high-glucose (4500 mg/L) Dulbecco’s Modified Eagle’s medium (DMEM), supplemented with 10% fetal bovine serum (FBS) and 100 μg/mL penicillin/streptomycin, under an atmosphere of 5% CO_2_ at 37 °C. Parasites were left to interact with the cells for 4 h and were then washed 3 times with phosphate saline buffer (PBS), pH 7.4, to eliminate unattached parasites. Then, 5–6 days post-infection, trypomastigotes were obtained from the supernatant, centrifuged at 4000× *g* for 15 min, and washed 3x with PBS. Subsequently, parasites were counted in a hemocytometer and frozen at −20 °C in 1 mL aliquots (10^5^ parasites/mL). These aliquots were later used for spiking GEB samples as required by each specific experimental design

### 4.2. DNA Extraction Methods

Silica column: Nucleotide extraction was performed using the High Pure Template Preparation kit (Roche Life Sciences (F. Hoffmann-La Roche Ltd., Basel, Switzerland), cat. no. 11796828001), as reported [12]. Briefly, 300 μL of guanidine–ethylenediaminetetraacetic acid (EDTA) blood (GEB) was added to 100 μL of binding buffer, 40 μL of proteinase K, and 4 μL of Exogenous Internal Positive Control (ExoIPC) synthetic DNA, according to the manufacturer’s instructions (TaqMan Exogenous Internal Positive Control Reagents (VIC Probe), Applied Biosystems (Thermo Fisher Scientific, Waltham, MA, USA), cat. no. 4308323), vortexed, and incubated at 70 °C for 10 min. Next, 100 μL of molecular-grade isopropanol was added to each sample, vortexed, and spun down for DNA precipitation. The silica column filter tubes were assembled into collection tubes, and samples were applied carefully onto the upper buffer reservoir of the filter tube and centrifuged for 1 min at 8000× *g*. The flow-through was discarded, and the filter was assembled into a new collection tube and subjected to successive washes with an Inhibitor Removal Buffer and wash buffer to remove inhibitors and contaminants. After the washing steps, filters were assembled into a new collection tube and centrifuged for 1 min at 15,000× *g* to remove any traces of remaining buffers. Lastly, the filter tube was inserted into a 1.5 mL microtube, where 100 µL of pre-warmed Elution buffer was added, and it was centrifuged for 1 min at 8000× *g* to elute the DNA. Each extraction included non-spiked GEB as the negative control. Eluted DNA was stored at −20 °C until further analysis.

Magnetic beads: Nucleotide extraction was performed using the MagMax DNA Multi-Sample Ultra 2.0 kit (Applied Biosystems, cat. no. A36570) using the KingFisher Duo Prime System (Thermo Scientific, Waltham, MA, USA) according to the manufacturer’s instructions. Briefly, KingFisher 96 deep-well plate (Thermo Fisher Scientific, Cat n. 95040450) and elution strips were prepared with the appropriate quantity of buffers and 300 µL of GEB samples and processed in the KingFisher automated extraction equipment under the ‘MagMAX_Ultra2_400 uL_DUO’ protocol. Each 12-well extraction batch had a non-spiked GEB as the negative control. DNA was eluted in 100 µL of elution buffer, transferred to 0.2 mL PCR microtubes, and stored at −20 °C until further analysis.

### 4.3. T. cruzi Load Quantification by Quantitative Real-Time PCR

Amplification of *T. cruzi* satDNA by qPCR was performed with 2X FastStart Universal Probe Master (Roche Diagnostics (F. Hoffmann-La Roche Ltd., Basel, Switzerland), cat. no. 4913957001) using specific primers Cruzi1 (5′-ASTCGGCTGATCGTTTTCGA-3′) and Cruzi2 (5′-AATTCCTCCAAGCAGCGGAT A-3′), both at 750 nM, and TaqMan probe Cruzi3 (6FAM–CACACACTGGACACCAA–NFQ–MGB) at 50 nM, in multiplex with the Exo-IPC or RNAse P targets, as follows: for EXO-IPC (TaqMan Exogenous Internal Positive Control Reagents, cat. no 4308321, Applied Biosystems), 2 μL of [10×] IPC Primers/probe mix was used. For RNAse P, a human endogenous DNA target (TaqMan RNase P Detection Reagents Kit, cat. no 4316831, Applied Biosystems), 1 μL [20×] of RNAse P Primers/probe mix was used. Also, 5 μL of DNA was loaded, and the final reaction volume was 20 μL [10,23]. Real-time PCR assays were carried out on the Applied Biosystems QuantStudio 3 real-time PCR system (Thermo Fisher Scientific) using the following cycling conditions: 50 °C for 2 min, 95 °C for 10 min, followed by 40 cycles at 95 °C and 58 °C for 1 min, where fluorescence was collected at the annealing/extension step after each cycle. The results were collected and analyzed in QuantStudio Design and Analysis Software v1.5.2 (Applied Biosystems, Thermo Fisher Scientific, Waltham, MA, USA). All samples were analyzed in duplicate, and the threshold was set at 0.01 or 0.02.

### 4.4. Patient and Blood Samples and Ethics Statement

For clinical validation, GEB samples were collected from 40 individuals, 30 of whom tested positive for CD by the conventional serology (two positive serological tests) and were being monitored at the outpatient clinic of the Evandro Chagas National Institute of Infectious Diseases (INI) at the Oswaldo Cruz Foundation (Fiocruz), Rio de Janeiro, Brazil. The remaining 10 individuals had negative serology for CD. To confirm the diagnosis, two independent commercial serological tests were utilized, both of which were simultaneously reactive and conducted according to the manufacturer′s instructions: indirect immunofluorescence (Imuno-con, WAMA Diagnóstica, São Paulo, Brazil) and enzyme-linked immunosorbent assay (ELISA) (Chagastest Recombinant ELISA, Wierner Lab., Rosario, Argentina). Patients with CD were from different regions of Brazil (northeast, southeast, midwest, and south) and infected with different *T. cruzi* genotypes or DTUs (TcII, TcV, TcVI, TcII + VI, TcII/TcVI, and TcII/TcV/TcVI) [30], presenting the indeterminate or chronic cardiac forms of CD [31]. Individuals with negative serology were from a non-endemic area in the state of Rio de Janeiro. All qPCR assays were performed in two technical replicates, and the final result was considered valid only when both replicates yielded concordant outcomes. Written informed consent forms were signed by all the study subjects. All samples were pre-existent at the time of the present study and were anonymized before being processed. This study was approved by the Ethics Committee of INI/Fiocruz (CAAE 0070.0.009.000-07, approved on 21 January 2008, according to the principles of the Declaration of Helsinki) [32].

### 4.5. Statistical Analysis

All data were analyzed with SigmaPlot v14.0 (Systat Software, Chicago, IL, USA) or GraphPad Prism 10 software (GraphPad, San Diego, CA, USA). An unpaired one-way ANOVA was used to determine whether there were any significant statistical differences between the samples.

## 5. Conclusion

In conclusion, our study highlights the value of the magnetic beads purification method in improving the molecular diagnosis of Chagas disease, particularly in samples with low parasitemia, as commonly observed in chronic patients. The method demonstrated higher extraction yields, superior DNA quantity and purity, and full compatibility with GEB samples, a significant advantage in Chagas disease diagnosis. This study offers a fresh perspective on nucleotide purification methods in Chagas disease diagnosis and paves the way for enhanced clinical practices in molecular laboratories. With its higher reproducibility and less resource-intensive nature, the magnetic beads purification method has the potential to revolutionize the diagnostic process, leading to a more efficient and cost-effective approach to managing Chagas disease. Further research could explore the broader applications of this method across various clinical settings and its potential benefits in diagnosing other parasitic diseases.

## Figures and Tables

**Table 1:** ExoIPC and *T. cruzi* satDNA Ct mean comparison between silica column and magnetic beads methods with the calculation of ΔCt.

Sample	Silica Column ^a^		Magnetic Beads ^a^		ΔCt ^b^
*T. cruzi* Quantity (Par. Eq./mL)	Ct Mean (ExoIPC)	Ct Mean (*T. cruzi* satDNA)	Ct Mean (ExoIPC)	Ct Mean (*T. cruzi* satDNA)	(Ct_SC_ − Ct_MB_)
10^4^	33.57 ± 2.76	16.48 ± 0.14	38.08 ± 0.41	15.17 ± 0.12	1.32
10^3^	30.52 ± 3.15	19.70 ± 0.12	30.97 ± 0.18	18.58 ± 0.16	1.12
10^2^	28.82 ± 0.11	23.93 ± 0.28	28.02 ± 0.04	22.24 ± 0.03	1.69
10^1^	29.02 ± 0.04	26.81 ± 0.14	28.77 ± 0.02	25.95 ± 0.01	0.85
10^0^	29.07 ± 0.20	32.05 ± 2.20	29.00 ± 0.10	27.19 ± 0.27	4.87
10^−1^	29.12 ± 0.14	32.78 ± 3.30	29.33 ± 0.34	34.51 ± 0.00	−1.73
10^−2^	29.57 ± 0.11	ND	29.15 ± 0.12	ND	-
10^−3^	28.87 ± 0.08	ND	28.97 ± 0.18	ND	-
10^−4^	28.61 ± 0.14	ND	29.00 ± 0.10	ND	-
10^−5^	28.57 ± 0.16	ND	28.88 ± 0.07	ND	-
10^−6^	28.68 ± 0.13	ND	28.96 ± 0.11	ND	-

^a^ Not detected. ^b^ ΔCt is shown as mean ± SD for each group.

**Table 2:** Precision of qPCR assays targeting *T. cruzi* satDNA. Samples were assayed in 33 technical replicates at 5.0, 2.5, and 1.25 Par. Eq./mL.

Parameter	Sample Concentration		
	5.0 Par. Eq./mL	2.5 Par. Eq./mL	1.25 Par. Eq./mL
	Silica column		
Positive samples	33/33 (100%)	32/33 (96.9%)	26/33 (78.8%)
Ct mean (±SD)	33.04 ± 2.07	33.89 ± 1.63	35.98 ± 2.04
Coefficient of variation (%)	6.28%	4.81%	5.66%
	Magnetic beads		
Positive samples	33/33 (100%)	33/33 (100%)	30/33 (90.9%)
Ct mean (±SD)	31.45 ± 1.26	32.73 ± 1.40	34.19 ± 2.20
Coefficient of variation (%)	4.02%	4.28%	6.4%

**Table 3:** qPCR results for DNA extracted from blood samples of individuals with positive or negative serology for Chagas disease.

DNA Extraction	Patient Samples (*n* = 40)			
	Silica Column		Magnetic Beads	
	qPCR+	qPCR−	qPCR+	qPCR−
CD-positive serology (*n* = 30)	11 (36.7%)	19 (63.3%)	11 (36.7%)	19 (63.3%)
CD-negative serology (*n* = 10)	0 (0%)	10 (100%)	0 (0%)	10 (100%)
Total	11	29	11	29

## Data Availability

The original contributions presented in this study are included in the article/Supplementary Materials. Further inquiries can be directed to the corresponding authors.

## Supplementary Materials

The following supporting information can be downloaded at: https://www.mdpi.com/article/10.3390/ijms26030937/s1.
