# Supplementary material for: Utility of Magnetic Bead-Based Automated DNA Extraction to Improve Chagas Disease Molecular Diagnosis
Source: Int J Mol Sci. 2025 Jan 23;26(3):937. doi: 10.3390/ijms26030937 (PMC11816604; doi:10.3390/ijms26030937)
Supplement: Supplementary file 1 [file ijms-26-00937-s001.zip › ijms-3375425-supplementary.pdf]

Tables

Table S1. Exo IPC and *T. cruzi* satDNA Ct mean comparison between silica column and magnetic beads methods with the calculation of ΔCt. ΔCt is shown as mean ± SD for each group.

**Sample**	**Silica Column**		**Magnetic beads**		**Δ Ct**
***T. cruzi* quantity (Par.Eq/mL)**	**Ct mean (Exo IPC)**	**Ct Mean (*T. cruzi* SatDNA)**	**Ct mean (Exo IPC)**	**Ct Mean (*T. cruzi* SatDNA)**	**(Ct $^{\text{Silica column}}$ – Ct $^{\text{Magnetic beads}}$)**
10^4	33.57 ± 2.76	16.48 ± 0.14	38.08 ± 0.41	15.17 ± 0.12	1.32
10^3	30.52 ± 3.15	19.70 ± 0.12	30.97 ± 0.18	18.58 ± 0.16	1.12
10^2	28.82 ± 0.11	23.93 ± 0.28	28.02 ± 0.04	22.24 ± 0.03	1.69
10^1	29.02 ± 0.04	26.81 ± 0.14	28.77 ± 0.02	25.95 ± 0.01	0.85
10^0	29.07 ± 0.20	32.05 ± 2.20	29.00 ± 0.10	27.19 ± 0.27	4.87
10^{-1}	29.12 ± 0.14	32.78 ± 3.30	29.33 ± 0.34	34.51 ± 0.00	-1.73
10^{-2}	29.57 ± 0.11	0 ± 0.00	29.15 ± 0.12	0 ± 0.00	0.00
10^{-3}	28.87 ± 0.08	0 ± 0.00	28.97 ± 0.18	0 ± 0.00	0.00
10^{-4}	28.61 ± 0.14	0 ± 0.00	29.00 ± 0.10	0 ± 0.00	0.00
10^{-5}	28.57 ± 0.16	0 ± 0.00	28.88 ± 0.07	0 ± 0.00	0.00
10^{-6}	28.68 ± 0.13	0 ± 0.00	28.96 ± 0.11	0 ± 0.00	0.00

Table S2. Amplification of *T. cruzi* SatDNA for silica column and magnetic beads extraction methods. Samples were assayed in 33 technical replicates and the percentage of positivity was calculated for each Par.Eq./mL concentration.

T. cruzi	**Silica Column**		**Magnetic Beads**	
**Par.Eq./mL**	**Amplification**	**Positivity (%)**	**Amplification**	**Positivity (%)**
20	33/33	100.00	33/33	100.00
10	33/33	100.00	33/33	100.00
5	33/33	100.00	33/33	100.00
2.5	32/33	96.97	33/33	100.00
1.25	26/33	78.78	30/33	90.90
0.625	10/33	30.30	24/33	72.72
0.3125	6/33	18.18	16/33	48.48
0.15625	4/33	12.12	12/33	36.36
0.078125	2/33	6.06	17/33	51.51
0.0390625	1/33	3.03	3/33	9.10
0.01953125	0/33	0.00	2/33	6.06

Table S3. Precision of qPCR assays targeting *T cruzi* SatDNA. Samples were assayed in 33 technical replicates at 5, 2.5, and 1.25 Par.Eq./mL.

Parameter	Sample concentration		
	**5 Par.Eq./mL**	**2.5 Par.Eq./mL**	**1.25 Par.Eq./mL**
**Silica column**			
**Positive samples**	33/33 (100%)	32/33 (96.9%)	26/33 (78.8%)
**Ct mean (± SD)**	33.04 ± 2.07	33.89 ± 1.63	35.98 ± 2.04
**Coefficient of variation (%)**	6.28%	4.81%	5.66%
**Magnetic beads**			
**Positive samples**	33/33 (100%)	33/33 (100%)	30/33 (90.9%)
**Ct mean (± SD)**	31.45 ± 1.26	32.73 ± 1.40	34.19 ± 2.20
**Coefficient of variation (%)**	4.02%	4.28%	6.4%

Figures

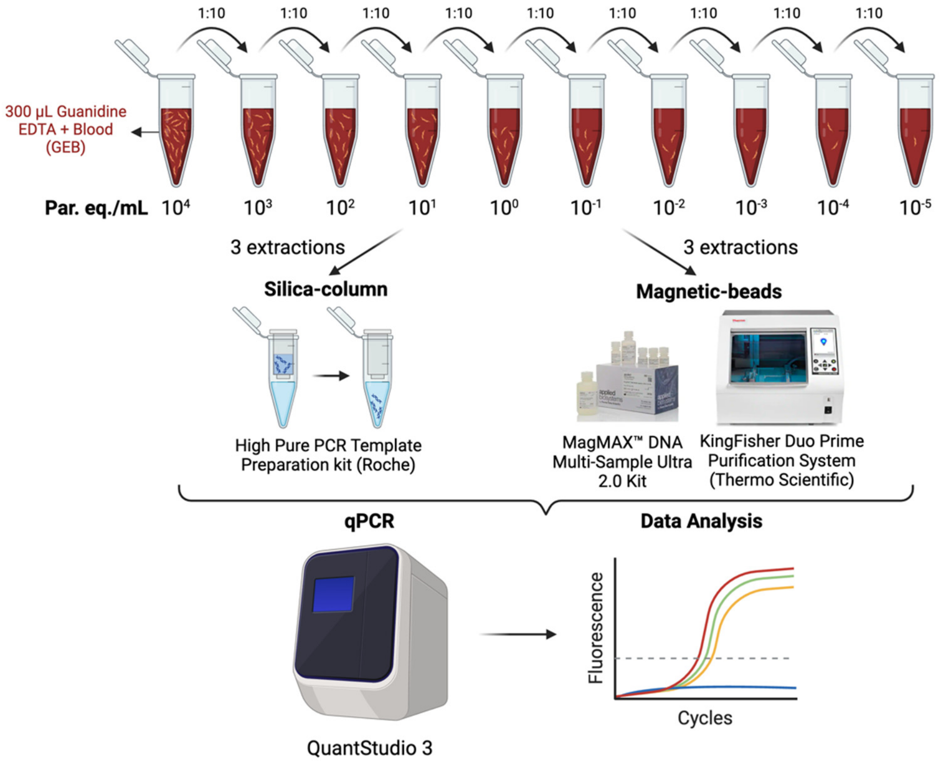

Supplementary Figure S1. Schematic representation of the *T. cruzi* satDNA assessment method. Aliquots of 300 mL GEB were spiked with varying concentrations of *T. cruzi* (ranging from 10^4 to 10^{-5} Par. Eq./mL) along with 4 mL ExoIPC as an internal positive control. GEB aliquots were extracted in 3 independent rounds using the silica column-based High Pure PCR Template Preparation Kit and the magnetic beads MagMAX DNA Multi-Sample Ultra 2.0 Kit, processed via the automated KingFisher Duo Prime purification system. The extracted DNA was then subjected to quantitative real-time PCR for *T. cruzi* satDNA quantification, ExoIPC, and RNAseP assessment using a TaqMan-based assay.

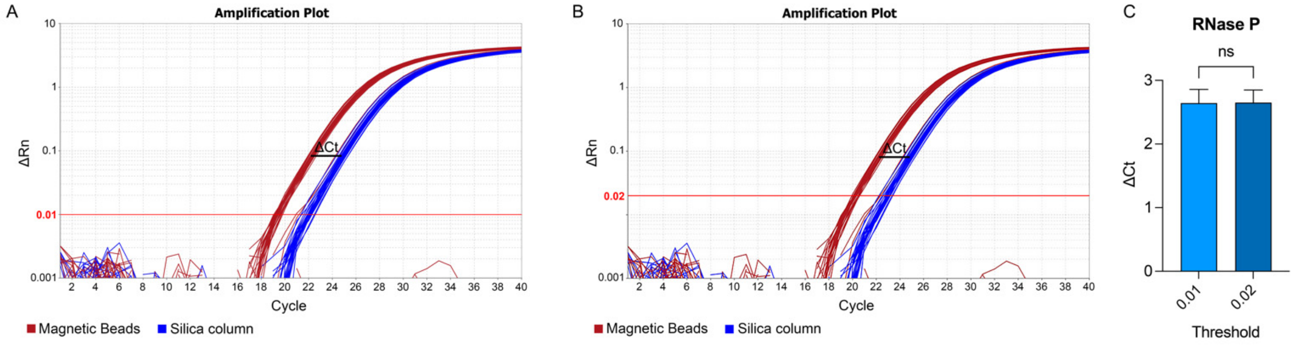

Supplementary Figure S2. Real-time quantitative PCR for RNAse P. RNAse P was assessed using the TaqMan detection system and quantified using a threshold of (A) 0.01 and (B) 0.02 to calculate the (C) Ct ($Ct_{SC} - Ct_{MB}$). Ct values are shown as mean ± SD for each group. Statistical significance was determined using one-way ANOVA with pairwise multiple comparisons (ns, non-significant).

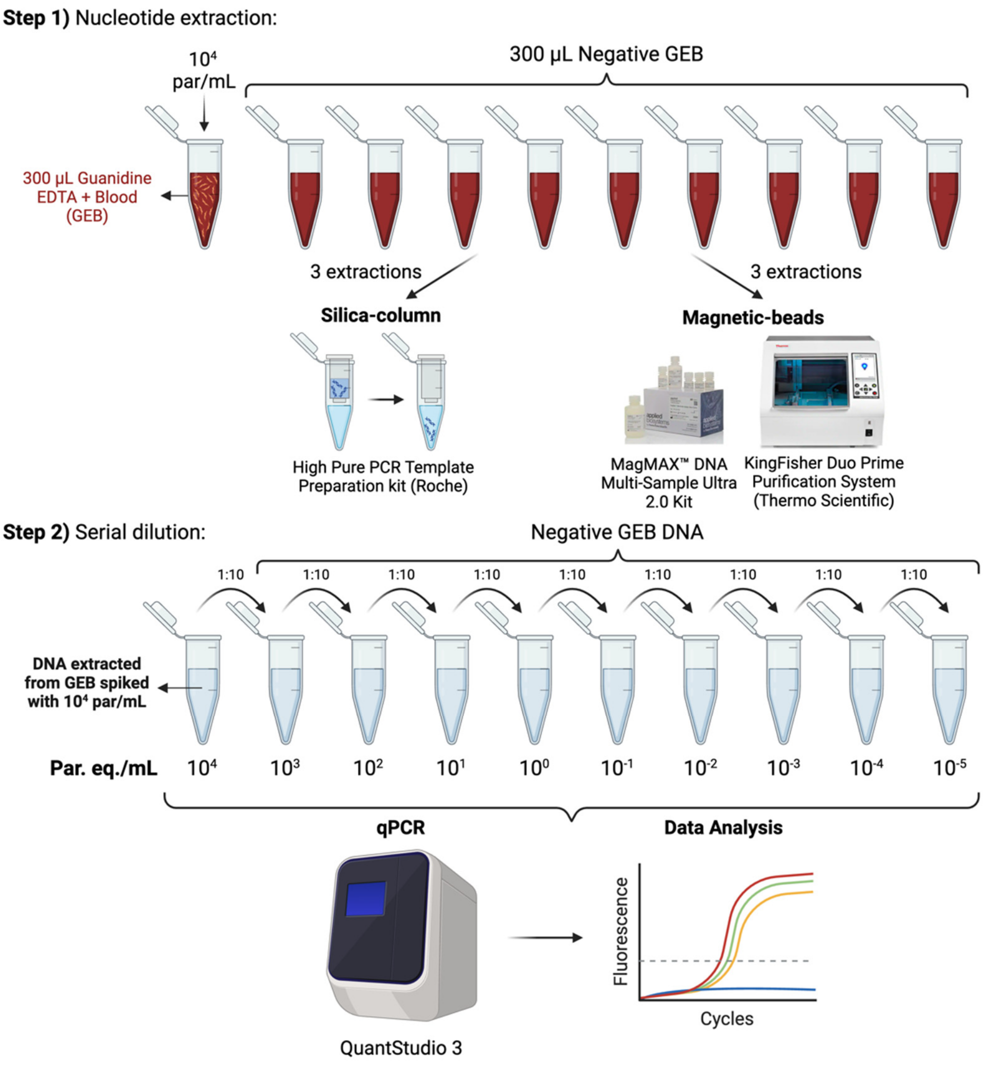

Supplementary Figure S3. Schematic representation of the *T. cruzi* satDNA assessment method for the standard curve. In the first step, a 300-µL aliquot of GEB spiked with 10^4 Par. Eq./mL, along with 9 aliquots of 300 µL non-spiked GEB (negative control), were extracted in 3 independent rounds using both the SC-based High Pure PCR Template Preparation Kit and the MB-based MagMAX DNA Multi-Sample Ultra 2.0 Kit and processed via the automated KingFisher Duo Prime purification system. In the second step, the extracted DNA from the GEB spiked with 10^4 Par. Eq./mL was subjected to a 10-fold serial dilution in non-spiked GEB DNA (ranging from 10^4 to 10^{-5} Par. Eq./mL) for both extraction methods. The diluted samples were then analyzed using quantitative real-time PCR for *T. cruzi* satDNA, ExoIPC, and RNAseP assessment with a TaqMan-based assay.

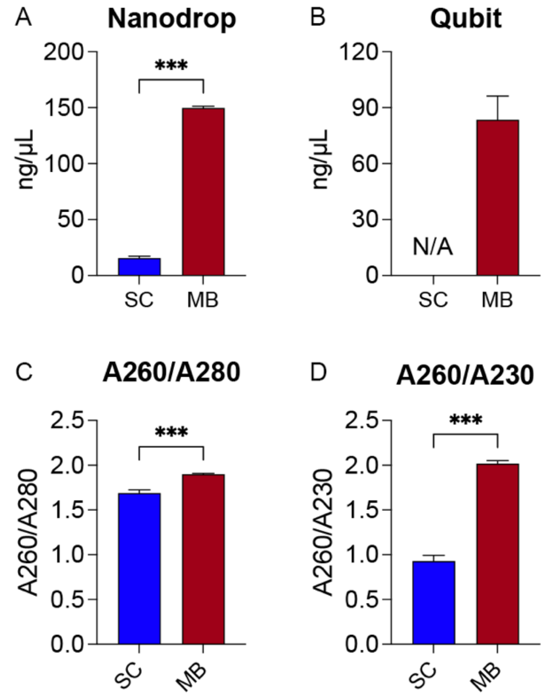

Supplementary Figure S4. DNA extraction yields and purity ratios for the silica-column kit and automated magnetic-bead purification system. DNA was extracted from GEB samples using either the silica-column-based kit (blue) or the automated magnetic-bead purification system (red) and quantified using (A) NanoDrop and (B) Qubit. Purity ratios were assessed through (C) 260/280 and (D) 260/230 measurements using NanoDrop. DNA concentration is presented as the mean ± SD for each group. Statistical significance was determined using one-way ANOVA with all pairwise multiple comparisons (***, $p < 0.001$).

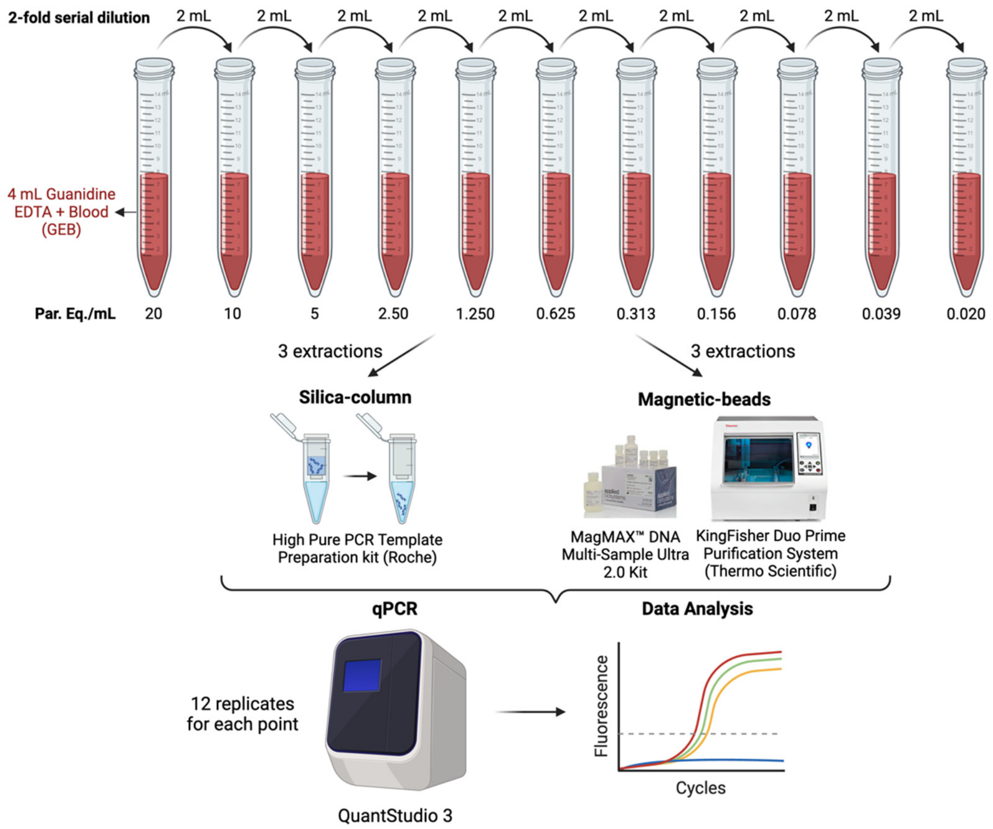

Supplementary Figure S5. Schematic representation of the *T. cruzi* satDNA assessment method for the limit of detection (LOD) assay. A 4-mL aliquot of GEB sample was spiked with 20 Par. Eq./mL and then subjected to a 2-fold serial dilution in 2-mL non-spiked GEB, ranging from 20 to 0.020 Par. Eq./mL. GEB aliquots were extracted in 3 independent rounds using both the SC-based High Pure PCR Template Preparation Kit and the MB-based MagMAX DNA Multi-Sample Ultra 2.0 Kit, processed via the automated KingFisher Duo Prime purification system, with a total of three extractions per method. The extracted DNA was analyzed by quantitative real-time PCR in 12 replicates for *T. cruzi* satDNA quantification and Exo IPC assessment using a TaqMan-based assay.
